# Type II secretion in *Yersinia*—a secretion system for pathogenicity and environmental fitness

**DOI:** 10.3389/fcimb.2012.00160

**Published:** 2012-12-14

**Authors:** Dominik von Tils, Inga Blädel, M. Alexander Schmidt, Gerhard Heusipp

**Affiliations:** ^1^Center for Molecular Biology of Inflammation (ZMBE), Institute of Infectiology, Westfälische Wilhelms-Universität MünsterMünster, Germany; ^2^Rhine-Waal University of Applied SciencesKleve, Germany

**Keywords:** type II secretion, pathogenicity, environmental fitness, *Yersiniae*, *Y. enterocolitica*

## Abstract

In *Yersinia* species, type III secretion (T3S) is the most prominent and best studied secretion system and a hallmark for the infection process of pathogenic *Yersinia* species. Type II secretion (T2S), on the other hand, is less well-characterized, although all *Yersinia* species, pathogenic as well as non-pathogenic, possess one or even two T2S systems. The only *Yersinia* strain in which T2S has so far been studied is the human pathogenic strain *Y. enterocolitica* 1b. Mouse infection experiments showed that at least one of the two T2S systems of *Y. enterocolitica* 1b, termed Yts1, is involved in dissemination and colonization of deeper tissues like liver and spleen. Interestingly, *in vitro* studies revealed a complex regulation of the Yts1 system, which is mainly active at low temperatures and high Mg^2+^-levels. Furthermore, the functional characterization of the proteins secreted *in vitro* indicates a role of the Yts1 machinery in survival of the bacteria in an environmental habitat. *In silico* analyses identified Yts1 homologous systems in bacteria that are known as plant symbionts or plant pathogens. Thus, the recent studies point to a dual function of the Yts1 T2S systems, playing a role in virulence of humans and animals, as well as in the survival of the bacteria outside of the mammalian host. In contrast, the role of the second T2S system, Yts2, remains ill defined. Whereas the T3S system and its virulence-mediating role has been intensively studied, it might now be time to also focus on the T2S system and its role in the *Yersinia* lifestyle, especially considering that most of the *Yersinia* isolates are not found in infected humans but have been gathered from various environmental samples.

## Secretion—A milestone in bacterial evolution

Bacteria have evolved since billions of years and have adapted to any niche present on our planet. Be it the hot and high-pressure environment of black smokers in the depth of the oceans, the high-salinity waters at a saltlake or the endurant ice of the permafrost, bacteria have found a way to populate nearly every spot on the earth (Boyd and Boyd, 1964; Abyzov et al., 1979; Corliss et al., 1979; Imhoff, 1986; Schrenk et al., 2003; Zhou et al., 2009). Although the mentioned microbes are highly specialized extremists and have adapted to their natural reservoirs during eons, it should be kept in mind that many of the bacteria in less exotic environments are also highly adaptable (Cases et al., 2003; Kotte et al., 2010). To ensure that they are using the adequate tools at the right time the microbes have to sense and to react to changing conditions in their environment. A simple change in temperature can trigger the transcription of hundreds of genes which allows the bacterium to adapt to the new conditions (Hurme and Rhen, 1998; Herbst et al., 2009). But bacteria not only react to their environment, they are also able to influence and recreate their habitat. By secretion of proteins like amylases, chitinases, proteases, or iron and manganese reducing molecules, bacteria can manipulate their surrounding and exploit whatever nutrient sources are present (Francetic et al., 2000; Ast et al., 2002; De Vrind et al., 2003; Lee et al., 2004). The process of secretion is so crucial for the bacteria that several secretion systems have evolved. Especially the secretion machineries of Gram-negative microbes have evolved to highly complex mechanisms.

Because of the three layers of cell wall structure, the secretion systems of Gram-negatives have to deliver their cargo over the cytoplasmic membrane, the periplasmic peptidoglycan layer and the outer membrane. So far six secretion systems have been characterized for Gram-negative bacteria (T1SS–T6SS) (Sandkvist, 2001a; Delepelaire, 2004; Henderson et al., 2004; Filloux et al., 2008; Fronzes et al., 2009; Hauser, 2009) and some of them (T3SS, T4SS, T6SS) are even able to penetrate an additional layer, namely the lipid membrane of their host. Particularly pathogenic bacteria are thus equipped with a weapon to directly transmit effector proteins into the host cells.

## *Yersinia* and T3SS

One of these sophisticated secretion systems is the T3SS, termed Ysc, of *Yersinia* (Cornelis, 2002). *Yersinia* belong to the family of *Enterobacteriaceae* and are, like all γ-proteobacteria, Gram-negative microbes. Although the genus of *Yersinia* comprises at least 16 species, the most famous ones are the three human pathogenic species. Among them are the origin of the plague *Y. pestis* and the gastrointestinal disease causing *Y. pseudotuberculosis* and *Y. enterocolitica*. The mentioned T3SS Ysc is a hallmark of all three human pathogenic *Yersinia* species and is encoded on a plasmid of ~70 kb, named pYV (plasmid associated with *Yersinia* virulence) (Cornelis et al., 1987; Cornelis, 2002). This plasmid comprises whole weaponry against host cells: along with the T3SS it encodes a variety of type III secreted effector proteins. These proteins are called Yops (*Yersinia* outer proteins) and due to their importance during infection, the Yops and the T3SS have been intensively investigated, and their manipulating function on the hosts immune system is well-characterized (Cornelis and Wolf-Watz, 1997; Trosky et al., 2008). However, far less is known about another T3SS, termed Ysa (*Yersinia* secretion apparatus), which is also only prominent in the chromosome of pathogenic *Yersinia* species. It is highly similar in *Y. pestis* and *Y. pseudotuberculosis* whereas the *Y. enterocolitica ysa* gene cluster shows a less similar gene composition. Recent studies show that the Ysa system is important for early infection and invasion of the epithelial M-cells in the Peyer's patches (Young et al., 1999; Venecia and Young, 2005).

Furthermore, a flagellar T3SS has been identified showing homologies to known *E. coli* and *Salmonella* systems. Its main components are located around the *inv* gene encoding Invasin. As the virulence-associated phospholipase YplA is secreted via this flagellar T3SS, it is suggested that it might also contribute to pathogenicity of *Yersinia* (Young and Young, 2002).

In comparison to the pathogenic representatives, there are only few studies that focus on the remaining species of the *Yersinia* genus. Whereas some of these species are considered as opportunistic human pathogens (*Y. bercovieri*, *Y. frederiksenii*, *Y. intermedia*, *Y. kristensenii*, and *Y. mollaretii)*, most of them are classified as apathogenic environmental species (*Y. aldovae, Y. aleksiciae, Y. massiliensis, Y. pekanenii*, *Y. rohdei*, and *Y. similis*) (Bercovier et al., 1984; Wauters et al., 1988; Ibrahim et al., 1993; Sulakvelidze, 2000; Sprague and Neubauer, 2005; Sprague et al., 2008; Chen et al., 2010; Murros-Kontiainen et al., 2011; Souza et al., 2011). Only the insect pathogenic *Y. entomophaga* and the species *Y. ruckeri*, which causes redmouth disease in rainbow trouts, are known for their infectious lifestyle (Willumsen, 1989; Tobback et al., 2007; Hurst et al., 2011).

## T2SS—An introduction

All *Yersinia* species, except for *Y. pestis*, seem to be able to survive as free-living organisms. Even *Y. enterocolitica* and *Y. pseudotuberculosis* are widespread in the environment and have been isolated from soil, water, plants and both animal, and human faeces (Massa et al., 1988; Merhej et al., 2008).

One of the secretion machineries that are normally associated with the interaction of free-living bacteria with their environment is the type II secretion system. Type II secretion systems (T2SS) are widely distributed among Gram-negative bacteria, but are especially prominent among the γ-proteobacteria. Many of the substrates secreted by T2SS are degradative enzymes and include, among others, proteases, phospholipases, and chitinases (Francetic et al., 2000; DebRoy et al., 2006).

**Figure 1:**
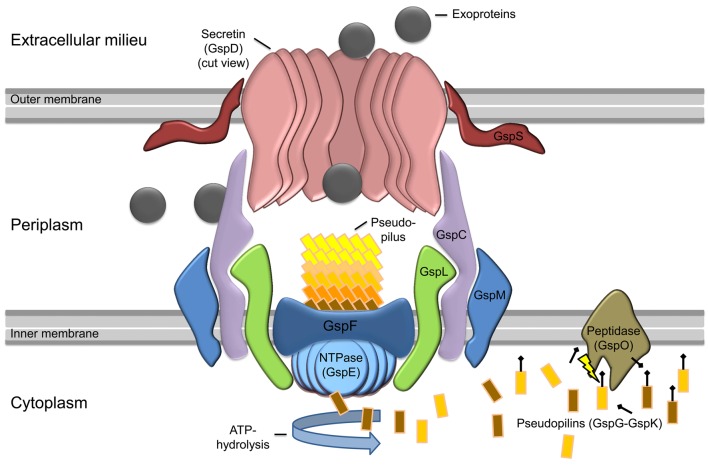
**Model of general type II secretion as proposed by Douzi et al. (2012).** Exoproteins (gray circles) are initially translocated over the inner membrane (IM) into the periplasmic space via the Sec or Tat pathway (not shown). The prepilin peptidase GspO processes the pseudopilins (GspG–GspK) by cleavage of the leader peptide. The cleaved pseudopilins are then assembled by ATP hydrolysis of the cytosolic NTPase (GspE) to form the pseudopilus. GspE is anchored to the inner membrane by GspL and thereby interacts with the IM-embedded GspF. In active state, the selection of secretion substrates is presumably performed by an interaction of GspC and GspD components. Then the appropriate exoproteins can enter the T2SS and are pushed into the extracellular milieu by the assembling pseudopilus. The exoproteins cross the outer membrane (OM) through the ring-formed channel (secretin) of GspD-subunits. In some species, GspS stabilizes GspD-subunits and prevents degradation of the secretin.

The T2SS is a multiprotein complex composed of 12–15 different proteins spanning the envelope of Gram-negative bacteria that is dedicated to the transport of secretion substrates through the bacterial outer membrane in a two-step process (Figure 1). First, either the Sec or the Tat pathway translocates the substrate through the cytoplasmatic membrane. The substrate is folded in the periplasm and is then transported through the outer membrane by the type II secretion machinery to reach its extracellular location (Douzi et al., 2012). The active process of the protein translocation is carried out by the assembly of pseudopili in the periplasm, a process energized by an inner membrane localized ATPase. This mechanism is quite similar to the *modus operandi* of a molecular machine that is involved in type-IV pilus (T4P) biogenesis. Indeed the T2SS shares an evolutionary relationship to the T4P biogenesis (Hobbs and Mattick, 1993; Peabody et al., 2003; Filloux, 2004). In the piston model for T2S, small subunits (pseudopilins) form a pseudopilus, which assembles from the basal part of the machinery, thereby “pushing” the substrates within the periplasmic space of the complex through the pore formed by the outer membrane secretin (Reichow et al., 2010). However, so far it is still unknown how the recognition of the specific secretion substrates is guaranteed. It is suggested that the assortment of substrates probably relies on a structural rather than a sequence motif (Filloux, 2004; Forest, 2008; Douzi et al., 2012; Korotkov et al., 2012).

Commonly the genes encoding the T2SS are found in clusters and their organization is well-conserved. They are often organized as a single operon, although variations occur at the 5′ and 3′ ends. The expression of the T2S genes is regulated by growth phase and environmental conditions in many bacteria, and quorum sensing is often involved in this process (Chapon-Hervé et al., 1997). However, the expression of substrates might be controlled differently. Therefore, regulation of secretion might occur at the level of transcription as well as at the level of substrate recognition (Sandkvist, 2001b).

Although the wide distribution of T2S in Gram-negative bacteria emphasizes the importance of the machinery, the knowledge about its function in human pathogens like *Y. enterocolitica* is still quite poor.

## T2SS in *Y. enterocolitica*—A double-sided sword?

Only recently, Iwobi et al. (2003) identified the first type II secretion gene cluster (*yts1C-S*) in *Y. enterocolitica* by representational difference analysis. Whereas this T2S was exclusively found in high-pathogenicity species, a second putative type II secretion gene cluster (*yts2*) seems to be distributed among nearly all *Y. enterocolitica* isolates. The Yts2 T2SS is discussed below.

Interestingly, the Yts1 secretion system was actually shown to be an effector on vertebrate virulence of *Y. enterocolitica*. Mouse infection experiments showed that an *yts1E* (ATPase)-deficient strain had a 100-fold reduced virulence regarding the infection of deeper tissues like liver and spleen. However, only infections via peroral application of the bacteria did lead to this effect, while it did not occur in mice intravenously inoculated with the bacteria. Due to the fact that the numbers of bacteria isolated from intestinal lavages and from Peyer's patches after infection were not significantly different between wildtype and *yts1E* infected mice, the authors concluded that this T2SS plays an important role after the invasion of M-cells. Therefore, it can be speculated that Yts1 plays an important role during a systemic infection by *Y. enterocolitica.* However, how Yts1 contributes to virulence during this stage of infection remains speculative from the study, also as the secretion substrates were not determined that might be responsible for the observed phenotype (Iwobi et al., 2003). In any case, the Yts1 system of *Y. enterocolitica* seems to play an important part during mammalian infection, but so far the majority of investigated T2SSs were described to be important for the environmental survival of the carrying bacteria. Only a few studies suggest a dual function of the T2SS in pathogenic bacteria, mediating both virulence capacity and a fitness factor for the survival in the natural reservoir. Among these candidates are the well-known pathogens *Legionella pneumophila* (Söderberg et al., 2004; Cianciotto, 2009; McCoy-Simandle et al., 2011) and *Vibrio cholerae* (Kirn et al., 2005; Stauder et al., 2012; Wong et al., 2012). Now, a recent study that investigated the regulation and the secretion of Yts1 *in vitro*, also points to a possible function of the Yts1 system for the survival of *Y. enterocolitica* in its environmental reservoirs (Shutinoski et al., 2010). Here, we therefore seek to highlight the recent findings on the two T2SSs (Yts1, Yts2) in *Y. enterocolitica*. In particular we will describe the distribution of homologous systems in *Yersinia* species and other groups of bacteria and we will discuss the indications for a possible dual function of Yts1 for both the pathogenicity and the environmental survival of *Y. enterocolitica*.

## Yts2 in *Y. enterocolitica*

Although little is known about the Yts2 secretion system and no secreted proteins could be identified so far, we like to summarize here all the available information about Yts2 first, before we discuss the more profound knowledge about the related Yts1 system. While Yts1 was discovered by representational difference analysis, the existence of the Yts2 T2SS in *Y. enterocolitia* was identified by *in silico* analysis due to high similarity to the previously discovered Yts1 system (Iwobi et al., 2003). Generally the *yts2* gene cluster corresponds to the common type II secretion model. However, compared to this standard model, the *yts2* gene cluster lacks the gene *gspS* and in several *Yersinia* species also *gspH* is absent (Table 1). Worth to mention is also the unexpected low homology of the putative GspM of the Yts2 system compared to GspM proteins in other T2SS. But the genomic localization of this putative protein, its calculated pI and its prediction as a bitopic membrane protein point to an equal functionality as suggested for known GspM proteins. GspM is indispensable for the secretion process and it is supposed to direct the localization of the inner membrane component GspL toward specific sites in the cell wall (Michel et al., 1998). The *gspS* gene is absent in all *yts2* clusters throughout the *Yersinia* species. However, in some bacteria it supports the incorporation of the secretin into the outer membrane and inhibits degradation of the GspD subunits (Hardie et al., 1996; Shevchik and Condemine, 1998). It should also be noted that in some species GspS seems to be substituted by a GspAB complex. Here, GspAB is suggested to mediate multimerization and transport of secretin subunits into the outer membrane (Ast et al., 2002). Furthermore, GspD subunits like the liposecretin HxcQ from *Pseudomonas aeruginosa* are even independent of GspS/GspAB and are able to self-pilot to the outer membrane by their N-terminal lipid anchor (Viarre et al., 2009). However, whether the lack of GspS in the Yts2 secretion system is counterbalanced by one of the mentioned mechanisms remains to be investigated. GspH is one of the five pseudopilins, which are thought to be part of the secretion pilus (Yanez et al., 2008). It is not clear yet if all pseudopilins are essential for the assembly of a functional secretion pilus or if the loss of *gspH* can be compensated for by the other four pseudopilins.

**Table 1:** **Occurrence of Yts1(-like) and/or Yts2 secretion systems and corresponding substrates in *Yersinia* and other bacterial species**.

	**Yts1**	**Yts2**
Expression conditions (in *Y. enterocolitica* O:8)	*In vivo:*	No extrinsic expression conditions identified so far
	Expression inferred from colonization defect associated with the lack of *yts1E* during experimental mouse infection	
	*In vitro:*	
	High expression: low temperatures (17°C) and high Mg^2+^-levels (20–50 mM) in LB medium and diverse minimal media. Activation of *pclR*, *yts1* genes and *chiY* transcription	
	Minimal expression: 37°C in LB and any other tested growth medium. Minimal transcription of *pclR*, *yts1* genes and *chiY*	
Regulators (in *Y. enterocolitica* O:8)	PclR: overproduction activates *chiY* transcription under high Mg^2+^-conditions (20–50 mM)	PypC: overproduction results in *pypC* transcription (autoregulation)
Known secretion substrates (in *Y. enterocolitica* O:8 strain 8081)	ChiY (YP_001007736.1)^*^	No secretion substrates known so far
	EngY (YP_001007806.1)^*^	
	YE3650 (YP_001007019.1)^*^	
Species harboring	*Y. enterocolitica* O:8	*Y. enterocolitica* O:8
Yts1(-like) and/or Yts2 secretion system	*Y. ruckeri* [-GspI]	*Y. ruckeri* [-GspI]
	*Y. aldovae*	*Y. enterocolitica* W22703 (O:9)
	*Y. mollaretii* [-YE3650]	*Y. pestis*
		*Y. pseudotuberculosis*
	Yts1-like secretion systems:	*Y. bercovieri* [-GspH]
	*E. amylovora* [-EngY; -YE3650]	*Y. frederiksenii* [-GspH]
	*E. tasmaniensis* [-EngY; -YE3650]	*Y. intermedia* [-GspH]
	*E. pyrifolia* [-EngY; -YE3650]	*Y. kristensenii* [-GspH; -PypC]
	*S. proteamaculans* [-EngY; -YE3650]	*Y. rohdei* [-GspH; -PypC]
	*P. aeruginosa* [-PclR; -GspC; -EngY; -YE3650]	
	*P. putida* [-PclR; -GspC; -EngY; -YE3650]	

[ ], Indicating missing T2S components or substrates. All Yts2 secretion systems of Yersiniae lack GspS components. An asterisk (

* ) indicates the NCBI Ref. Seq. in parenthesis.

Strikingly, in *Y. enterocolitica* and in all other *Yersinia* species both T2SS are preceded by regulators (Shutinoski et al., 2010). In the case of Yts2, the corresponding gene is termed *pypC*. However, so far it was not possible to identify any *in vitro* condition that resulted in an elevated transcription rate of the *yts2* genes or of *pypC*. Only the artificial overproduction of the transcriptional regulator PypC led to an increase of the *yts2* transcription rate and elevated the expression of the *yts2C* gene by a factor of four, indicating that the *yts2* genes are indeed controlled by the cognate PypC regulator. Together with PypA and PypB, PypC is part of a complex regulatory network controlling transcription of the *hreP* gene encoding a protease that is specifically induced during infection (Young and Miller, 1997; Wagner et al., 2009). Therefore, Yts2 expression is linked to virulence and *in vivo* expression.

The results of a reverse transcription analysis indicated that a weak transcription of *yts2* is induced at lower temperatures (27°C) but is further reduced at mammalian host specific temperatures (37°C) (Iwobi et al., 2003). Yet an *in vivo* activation of Yts2 at 37°C is not necessarily excluded. As PypC is part of the regulatory system that controls the expression of the *in vivo* expressed HreP protease, it can be speculated if also the Yts2 system needs host specific activation factors and is therefore co-expressed with HreP in the mammalian host microenvironment (Wagner et al., 2009). Furthermore the *in vivo* environment might also be essential for the transcription and secretion of the substrates of Yts2.

Regarding the distribution of the T2SS in *Yersinia* species, *in silico* analyses showed that the *yts2* secretion cluster is prominent in nearly all sufficiently sequenced *Yersinia* species (Table 1). Only *Y. aldovae* and *Y. mollaretii* lack the *yts2* gene cluster. Furthermore, there is a highly conserved genetic linkage of the *pypC* analogues with the T2SS clusters so that all human pathogenic and most opportunistic pathogenic *Yersinia* species (*Y. pestis*, *Y. pseudotuberculosis*, *Y. enterocolitica, Y. frederiksenii, Y. intermedia*, and *Y. kristensenii*) carry this transcriptional regulator.

## Yts1 in *Y. enterocolitica*

The genes of the Yts1 secretion system are chromosomally encoded in a *plasticity zone* which comprises several virulence associated factors including another T3S system, termed Ysa (Young and Young, 2002; Thomson et al., 2006; Young, 2007). The 13 core components (*yts1C-M, yts1O, yts1S*) of the machinery are transcribed as a single operon, with the exception of the *yts1S* gene that is located on the antisense strand. Immediately downstream, the *yts1* operon is followed by the *chiY* gene, which was identified to encode one of the main secretion substrates of Yts1 (Shutinoski et al., 2010). Similar to the *yts2* cluster, a regulator is encoded immediately 5′ of the *yts1* operon. Due to its similarity to the PypC regulator on amino acid (aa) level (44%) the Yts1 homologue was termed PypC-like regulator (PclR).

However, the investigations on the regulation of Yts1 expression led to conflicting results. While Iwobi et al. (2003) performed real-time PCR experiments that, according to the mouse infection model, showed that *in vitro* transcription of *yts1* genes was higher at 37°C than at 27°C, *in vitro* studies of the *yts1* expression performed by Shutinoski et al. (2010) created a contradicting picture (Shutinoski et al., 2010). Here, the transcription of the operon and functional secretion of newly discovered substrates were elevated at lower temperatures (17°C) and high Mg^2+^-ion concentrations (20 mM). With rising temperatures, transcription of *yts1* genes as well as secretion of substrates were reduced and were nearly undetectable at 37°C. This conflicting finding might be caused by the use of different *Y. enterocolitica* 1b strains. Whereas Iwobi et al. (2003) used the *Y. enterocolitica* serotype O:8 strain WA-314, Shutinoski et al. (2010) performed their experiments with the closely related O:8 strain 8081. Interestingly the WA-314 genome exhibits a novel IS-element (IS1330) upstream of the Yts1 secretion cluster, whereas it is lacking in proximity to the Yts1 operon in the 8081 strain. It can therefore be speculated that this insertion might be the cause of the differential regulation between the two strains. This has to be proven in future experiments.

To further investigate the regulation of Yts1 expression, Shutinoski et al. (2010) tested the impact of the regulator PclR on *yts1* transcription. Therefore they induced PclR overproduction (PclR-OP) from a plasmid under the control of an inducible promoter. High PclR levels did not lead to an increased transcription of genomic *pclR* nor to elevated levels of *yts1C* transcripts, but PclR-OP caused *chiY* transcription levels to rise and thereby increased the amount of ChiY protein secreted. The regulation of *chiY* transcription is therefore not only dependent on temperature and Mg^2+^-concentrations but is also affected by PclR. Thus, the function of PclR seems to be the coordination of expression of the Yts1 secretion system with its cognate substrate. However, PclR is not sufficient as a positive factor alone, but is only effective under high Mg^2+^-conditions (Shutinoski et al., 2010). Whether this effect is due to the interaction of PclR with a yet unknown regulator or whether the Mg^2+^-ions have a direct impact on PclR-binding to the promotor region of the *chiY* gene, remains unknown. In contrast to the effect of high Mg^2+^-levels on gene expression, low Mg^2+^-concentrations are well-studied in Gram-negative bacteria (Stan-Lotter et al., 1979; Smith and Maguire, 1993, 1998). Several studies imply a role of the Mg^2+^-sensing PhoP/Q system in this regulatory process. In *Salmonella enterica*, the membrane spanning sensor PhoQ was shown to activate the cytosolic PhoP molecule which subsequently acts as a transcription factor regulating the expression of numerous genes-dependent on Mg^2+^-availability (Shin and Groisman, 2005). This prompted us to investigate the effect of a *phoP* mutation on Yts1-mediated T2S. Interestingly, a *Y. enterocolitica phoP* mutant strain exhibited a reduced ability to activate *chiY* transcription (Seekircher, 2010). It is therefore supposed that PhoP could play at least a partial role in *chiY* regulation in dependency of Mg^2+^-concentrations.

**Figure 2:**
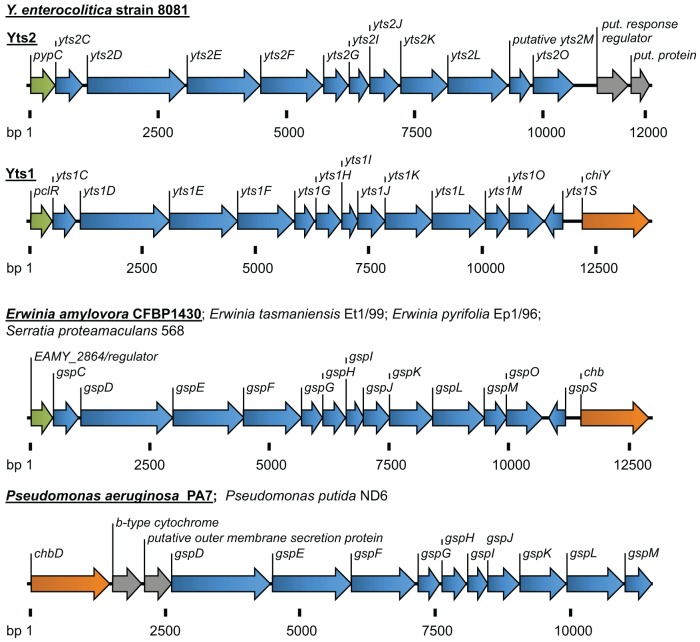
**Overview of the T2SSs Yts2 and Yts1 in *Y. enterocolitica*.** For comparison, T2SSs of *Erwinia, Serratia*, and *Pseudomonas* species are depicted. The size of gene clusters is indicated as basepairs (bp). Coloration of genes according to the (putative) function: transcriptional regulators (green), T2S components (blue), carbohydrate-binding molecules (orange), b-type cytochrome and proteins with unknown function (gray).

In addition, other (unknown) factors seem to be involved in this complex regulation, as there has to be a mechanism that coordinates the temperature dependency of the system. In any case, the genes for the regulator *pclR*, the *yts1* operon and the secretion substrate *chiY* seem to be coupled genetically as well as functionally. *In silico* analyses even show that they form an entity that is probably transmitted via horizontal gene transfer (Figure 2) and homologues of this entity can be found in several other bacteria.

However, beside the mentioned ChiY, other substrates of the *Y. enterocolitica* Yts1 T2SS have been identified (EngY and YE3650), but their expression and secretion seems to be regulated independently (Shutinoski et al., 2010). Both, *engY* as well as *ye3650* are transcribed constitutively and independent of PclR or Mg^2+^-concentration. Furthermore, they are not genetically linked to the *yts1* operon. While the transcription of the genes is constitutive, the secretion of their protein products is dependent on Yts1 expression. Therefore, the regulation of EngY and YE3650 secretion is taking place on protein level; once a functional Yts1 T2SS is established, all so far identified substrates can be secreted.

Taken together, a lot more information could be unraveled for the regulation and function of the Yts1 secretion system in comparison to the Yts2 system in *Y. enterocolitica*. However, as the gene clusters of Yts1 and Yts2 are highly similar on aa level and are both preceded by closely related transcriptional regulators (*pclR* and *pypC*, respectively), a cross-talk between these two secretion systems might be possible. Despite these similarities, no cross-talk has been determined so far. For example, PclR-OP did not enhance the transcription of the Yts2 genes nor did it trigger any secretion by the Yts2 apparatus. Vice versa, PypC-OP did not have any impact on the Yts1 system. Furthermore, *yts1*-activating conditions like low temperature (17°C) and high Mg^2+^-levels (20 mM) did not affect *yts2* regulation (Shutinoski and von Tils, unpublished data).

## Yts1—secretion substrates

The three secretion substrates of the Yts1 T2SS of *Y. enterocolitica* that are known today have been identified by Shutinoski et al. (2010). They compared the supernatants of wt and *yts1E- (ATPase-)* deletion strains and identified the mentioned secretion substrates ChiY (YE3576), EngY (YE2830) and YE3650 (Shutinoski et al., 2010). *In silico* analysis further confirmed that all three proteins have a typical N-terminal signal sequence.

The secreted protein ChiY is predicted as an N-acetylglucosamine-binding molecule. The 53 kDa protein harbors two chitin-binding domains and in accordance showed positive binding capacities in a chitin-beads assay (Shutinoski et al., 2010). ChiY is closely related to other N-acetylglucosamine-binding proteins like the *V. cholerae* colonization factor GbpA (40% similarity on aa level). GbpA was shown to be required for efficient intestinal colonization and to mediate attachment to human epithelial cells as well as to zooplankton (Stauder et al., 2012). The authors of the investigation suggest that GpbA is important for the survival of *Vibrio* species in their natural aquatic reservoir. They assume that GbpA's original function is the zooplankton attachment of *V. cholerae* and that due to similar sugar residues exposed by mammalian epithelial cells GbpA also became a virulence factor for vertebrate infection. As ChiY in *Y. enterocolitica*, GbpA is also secreted by the T2SS of *V. cholerae* (Kirn et al., 2005). Considering that *Yersiniae* are also found in aquatic habitats and regarding that *Y. enterocolitica* like *V. cholerae* infects the mammalian host via the intestine, it can be speculated that ChiY's function is of comparable importance for *Y. enterocolitica* as GbpA for *V. cholerae* (Bottone and Mollaret, 1977; Massa et al., 1988).

The second Yts1 secretion substrate, EngY (100 kDa) does also contain a chitin-binding and an additional carbohydrate-binding domain. Both are located in the C-terminal part of the molecule. Indeed those domains were shown to be functional and mediate EngY-binding to chitin-beads (Shutinoski et al., 2010). In addition, the amino acid sequence of EngY exhibits a domain of the M60-like superfamily whose members are related to the enhancin peptidase family. The enhancin peptidases are zinc-metalloproteases, which are encoded and carried by specific baculoviruses (Wang and Granados, 1997). In *Lepidoptera* those enhancin peptidases bind and degrade insecticidal mucin at the surface of insect midgut epithelium cells. Thus, these proteases support epithelium disruption and enhance the baculovirus infection in the insects. The closely related M60-like proteases have recently been associated with the successful colonization of both the invertebrate gut and of vertebrate mucosal surfaces by pathogenic and apathogenic microbes (Nakjang et al., 2012). The carbohydrate-binding module of EngY is in other enhancins supposed to specify the substrate of the intrinsic zinc-metalloprotease. According to this information it can be suggested that EngY's C-terminal domains mediate the attachment of the protein to specific carbohydrate-containing surfaces which are then degraded by its zinc-metalloprotease activity. However, until now, a catalytic activity of EngY has not been detected and it remains to be shown if the protein binds only to chitin-containing surfaces as found in insects or if it is also important for mucin-binding during vertebrate infection.

Also the third secreted protein, YE3650, can be associated with carbohydrate-binding. YE3650 has a molecular mass of about 72 kDa. It contains parts of a predicted pectate lyase superfamily domain. Pectate lyases are plant virulence factors that are typically encoded by plant pathogens like *Erwinia chrysanthemi* (Henrissat et al., 1995; Xie et al., 2012). Pectate lyases are depolymerizing enzymes that degrade the plant's pectate components and lead to cell death and tissue maceration. However, the putative pectate lyase domain of YE3650 is rather short (79 aa) and is annotated as a partial coding sequence missing a stop codon. Therefore it is questionable whether it represents a functional catalytic structure. A lot of pectate lyase family proteins are known to be carbohydrate-binding, but a specific binding substrate of YE3650 could not yet be identified. Anyhow, the idea that a functional pectate lyase could guarantee a source of energy from plant cells for *Y. enterocolitica*'s environmental survival is exciting, although quite speculative, especially as *Yersiniae* have never been associated with soft-rottening of plants. Nevertheless, it might be worth to test whether a glycan-binding screen with YE3650 shows potential attachment of this protein to plant associated surfaces. As many Gram-negative enteric pathogens like *Salmonella* or *E. coli* (EHEC) have been found associated with vegetables (Islam et al., 2005; Barak et al., 2011), it might also be possible that plant adhesive and degrading proteins are important for the intestinal colonization of herbivores.

## Yts1—BLAST analyses of the *yts1* system and secretion substrates within the *Yersinia* genus

The Yts1 T2SS seems to be present in all high-pathogenicity *Y. enterocolitica* species (serotypes O:8, O:13, O:20, O:21) but not in low-pathogenic *Y. enterocolitica* isolates (e.g., O:3, O:9). At least, none of the *yts1* probes hybridized to low pathogenicity *Y. enterocolitica* DNA during Southern blot experiments (Iwobi et al., 2003). In contrast, BLAST analyses show that some environmental *Yersinia* species, namely *Y. ruckeri*, *Y. aldovae*, and *Y. mollaretii* carry a Yts1 system. Although so far not annotated, the BLAST analysis also showed that the three species carry the complete set of genes encoding a functional Yts1 system. All the remaining sequenced *Yersinia* species, including *Y. pestis* and *Y. pseudotuberculosis*, have only one T2S system encoded in their genome and due to similarity on aa level, this can be considered a Yts2 homologue. Until a complete genomic sequence is available, no statement can be given about the existence of T2SSs in *Y. entomophaga*, *Y. aleksiciae*, *Y. massiliensis*, *Y. similis* and *Y. pekanenii*.

However, in all Yts1-positive bacteria, except for *Y. aldovae*, the *yts1* gene cluster is preceded by a gene encoding a putative cytochrome B and at its 3′ end is followed by gene homologues of the N-acetylglucosamine-binding protein ChiY.

Concerning the secretion substrates for the Yts1 system, *in silico* analyses revealed that the distribution of the secreted proteins ChiY, EngY and YE3650 is besides *Y*. *enterocolitica* limited to the species of *Y. aldovae*, *Y. mollaretii* and *Y. ruckeri*. The ChiY homologues in these species exhibit an identity between 76 and 73% compared to the *Y. enterocolitica* protein. Similar identities (79–73%) were also found for the EngY protein. While *Y. mollaretii* does not possess a *ye3650* gene, *Y. ruckeri* and *Y. mollaretii* encode a YE3650 homologue with 80 and 78% identity on aa level, respectively.

## T2S—function in other bacteria

T2SSs are widespread among Gram-negative proteobacteria and they are reported to feature a broad range of tools for the microbes. On the one hand, the secreted substrates function to exploit nutrient and energy sources to survive in environmental niches. Here, crucial functions are mediated by type II secreted proteins that for example degrade biopolymers (e.g., amylases, pectin lyases, chitinases, proteases, lipases, nucleases etc.), oxidize manganese (*Pseudomonas putida*) (De Vrind et al., 2003), reduce iron and manganese (*Shewanella oneidensis*) (DiChristina et al., 2002), bind chitin (*P. aeruginosa*) (Folders et al., 2000) or secrete levansucrose (*Gluconacetobacter diazotrophicus*) (Arrieta et al., 2004). On the other hand, studies show that T2S also plays an important role during host infections by pathogenic bacteria (Cianciotto, 2005), which might also be considered an environmental niche. The assumption of an important role of type II secretion in bacterial pathogenesis is based on several observations: First of all, many mammalian, fish, plant, and insect pathogens are equipped with either one or even two T2SSs and several studies showed that a genetically impaired T2SS or mutation of its secretion substrates led to a significant decrease in virulence of the bacteria in mouse models (Iwobi et al., 2003; Rossier et al., 2004; Ho et al., 2008; Jyot et al., 2011). Furthermore, secreted proteins that degrade or adhere to biopolymers can often also target host cell structures and are therefore known to be potent virulence factors. An example of a type II-secreted virulence factor is the zinc metalloprotease StcE of enterohaemorrhagic *E. coli* (EHEC). StcE cleaves the host protein C1-esterase inhibitor, mucin, and also glycoprotein 340. The degradative function of StcE on extracellular matrix components was associated with a threefold increase in EHEC intimate adherence to host cells (Grys et al., 2005). Also some exotoxins are secreted via T2S. For instance, the cholera toxin of *V. cholerae* and the exotoxin A of *P. aeruginosa* are type II-secreted factors that provoke strong impairment of host cells during infection (Sandkvist et al., 1997; Ball et al., 2002).

A T2SS that promotes both, environmental survival and pathogenic potency is described for *L. pneumophila.* Indeed, this T2SS (known as Lsp) was shown to dampen the cytokine response of macrophages and epithelial cells in the lungs of infected mice (McCoy-Simandle et al., 2011). Lsp was also the first example of a T2SS that is important for the intracellular replication of bacteria (Hales and Shuman, 1999; Rossier et al., 2004). At least 25 factors are secreted by the *Legionella* T2SS, among them the chitinase ChiA, which was associated with persistence of the bacteria in the lung (DebRoy et al., 2006). In addition to the pathogenic properties of Lsp, the same T2SS is essentially facilitating growth of *L. pneumophila* at low temperatures which resemble conditions of an environmental habitat (Söderberg et al., 2004). Regarding the mentioned properties of the type II-secreted proteins in *Y. enterocolitica*, it would not be surprising, if also the T2SSs of *Yersiniae* possess a dual function mediating both environmental survival and virulence capacities.

Another fact that indicates an environmental importance of Yts1, is the distribution of similar T2SSs in other bacterial species. The same linkage of an N-acetylglucosamine-binding protein to a 5′-encoded Yts1-like secretion system can be found in plant pathogens and symbionts like *Erwinia amylovora, E. tasmaniensis, E. pyrifoliae* and in *Serratia proteamaculans* (Figure 2). Strikingly the gene clusters in these bacteria are also preceded by a PclR-like transcriptional regulator. The close proximity of genes similar to ChiY and a Yts1-like secretion system was also observed in *P. aeruginosa* and *P. putida.* Here, the N-acetylglucosamine-binding protein is encoded 5′ of the T2SS and the PclR-like regulator gene is missing. Instead, the cluster is preceded at its 5′ end by a gene encoding a putative cytochrome B561. Interestingly, the linkage of a cytochrome-encoding gene with the Yts1 T2SS has been observed several times in bacterial species that carry the T2SS operon. It is not known whether this association is due to a preferred insertion site for horizontally acquired genes or if cytochrome B is actually playing a functional role in type II secretion.

Anyway, the comparison of the *Y. enterocolitica* Yts1 secretion system with other T2SSs gives a lot of evidences that this machinery might originate from bacteria with plant and insect habitats. Nevertheless, the properties of the secreted factors might also mediate attachment or even degradation of mammalian host surfaces and therefore could have evolved to be also important as virulence factors during *Yersinia* infection.

## Summary and outlook

In *Yersinia* species the T3S system and its role during infection has been intensively studied and great progress has been achieved understanding the details. In contrast, the recently discovered T2S systems and their importance in pathogenesis and/or environmental survival of the *Yersiniae* are still poorly understood. Therefore the possibility of a dual function and the versatile regulation of the Yts1 T2S system should be further analyzed in future studies.

While some of the secrets of the Yts1 system have been unraveled, the Yts2 T2SS, although prominent in nearly all *Yersinia* species, remains mysterious with respect to possible secretion substrates, and its place of action. So far, only artificial overexpression of its cognate transcriptional regulator, PypC, revealed a dependency of *yts2* transcription on its 5′-regulator and the linkage to the Pyp regulatory network. Thus, *in vitro* and/or *in vivo* conditions activating expression of this system need to be identified. This might also lead to the discovery of the cognate secretion substrates.

For Yts1 a lot more is known. It was shown that Yts1-dependent secretion is important for dissemination of the highly pathogenic *Y. enterocolitica* 1b strain into deeper tissues in a mouse infection model, but it is unclear whether the secreted factors are similar to those found in *in vitro* experiments. Similarity of the *in vitro* secretion substrate ChiY to the N-acetylglucosamine-binding protein GbpA of *V. cholerae* indicates that ChiY might be a factor affecting both, mammalian infection and environmental survival. Interestingly, activation of Yts1 during mouse infection at 37°C is in contrast to a repressed transcription of *yts1* genes *in vitro* at this temperature. Remarkable is also the fact that the gene composition with the transcriptional regulator *pclR* at the 5′ end of the *yts1* operon and the gene for the major secreted protein ChiY at its 3′ end can also be found in several plant pathogens and symbionts like *E. amylovora* or *S. proteamaculans*. For many of the plant-associated microbes it is not only known that they interact with their host organism but that they also use insects as vectors. *E. amylovora* for example has been shown to be dispersed by honey bees (Johnson, 1993). If a possible secretion of chitin-binding proteins like ChiY could eventually enhance the adhesion of *Erwinia* to insects and thereby facilitate the dispersal of the bacteria has not been investigated so far. However, for the *Yersinia* Yts1 system the results of the BLAST analyses and the predicted functions of the secreted substrates lead to the speculation that its importance in the environment lies somewhere at the intersection between plant and insect interaction. Further studies will show whether a comparable regulation of the Yts1 system and similar secreted factors as in *Y. enterocolitica* 1b can also be detected in plant symbiotic or plant pathogenic bacteria. Additionally, plant and insect models may be tested for adherence, uptake, or infection by *Yersinia* species. A lot of questions regarding the T2S system in *Yersinia* species currently remain open and future answers might not only enlighten the role of T2S in pathogenesis and environmental survival of *Yersinia* but they could also contribute to the understanding of T2S in plant pathogens and symbionts.

Evolutionary success of a human pathogenic bacterium is not only determined by its virulence potential, but also by its fitness outside of the human host. In both processes, the T2SSs of *Yersinia* might play an important role. The presented data indicate that the Yts1 T2SS is widespread in other non-human pathogenic bacteria. This gives interesting insights into bacterial evolution and adaptation of a T2SS from an environmental fitness factor to a virulence factor. Studying the complex transcriptional regulation of Yts1 opens up new clues on horizontal gene transfer and the integration of acquired genes and operons into existing regulatory networks.

### Conflict of interest statement

The authors declare that the research was conducted in the absence of any commercial or financial relationships that could be construed as a potential conflict of interest.
